# Heterozygous CDKL5 Knockout Female Mice Are a Valuable Animal Model for CDKL5 Disorder

**DOI:** 10.1155/2018/9726950

**Published:** 2018-05-27

**Authors:** Claudia Fuchs, Laura Gennaccaro, Stefania Trazzi, Stefano Bastianini, Simone Bettini, Viviana Lo Martire, Elisa Ren, Giorgio Medici, Giovanna Zoccoli, Roberto Rimondini, Elisabetta Ciani

**Affiliations:** ^1^Department of Biomedical and Neuromotor Sciences, University of Bologna, Bologna, Italy; ^2^Department of Medical and Clinical Sciences, University of Bologna, Bologna, Italy

## Abstract

CDKL5 disorder is a severe neurodevelopmental disorder caused by mutations in the X-linked CDKL5 (cyclin-dependent kinase-like five) gene. CDKL5 disorder primarily affects girls and is characterized by early-onset epileptic seizures, gross motor impairment, intellectual disability, and autistic features. Although all CDKL5 female patients are heterozygous, the most valid disease-related model, the heterozygous female *Cdkl5* knockout (*Cdkl5* +/−) mouse, has been little characterized. The lack of detailed behavioral profiling of this model remains a crucial gap that must be addressed in order to advance preclinical studies. Here, we provide a behavioral and molecular characterization of heterozygous *Cdkl5* +/− mice. We found that *Cdkl5* +/− mice reliably recapitulate several aspects of CDKL5 disorder, including autistic-like behaviors, defects in motor coordination and memory performance, and breathing abnormalities. These defects are associated with neuroanatomical alterations, such as reduced dendritic arborization and spine density of hippocampal neurons. Interestingly, *Cdkl5* +/− mice show age-related alterations in protein kinase B (AKT) and extracellular signal-regulated kinase (ERK) signaling, two crucial signaling pathways involved in many neurodevelopmental processes. In conclusion, our study provides a comprehensive overview of neurobehavioral phenotypes of heterozygous female *Cdkl5* +/− mice and demonstrates that the heterozygous female might be a valuable animal model in preclinical studies on CDKL5 disorder.

## 1. Introduction

Cyclin-dependent kinase-like 5 (CDKL5) disorder (OMIM no. 300203) is a severe neurodevelopmental disorder caused by mutations in the X-linked *CDKL5* gene. Primary clinical features include early-onset intractable epileptic seizures, gross motor impairment, severe intellectual disability, and autistic-like features [1–4]. The majority of CDKL5 patients are heterozygous females carrying missense, nonsense, splice, or frameshift CDKL5 gene mutations or a genomic deletion [5]. Due to the different *CDKL5* mutations and the variable X-chromosome random inactivation (XCI) in females, the phenotypic spectrum of the disease spans from milder forms—which include the possibility of autonomous walking and less severe epilepsy that is amenable to control—to severe forms featuring intractable seizures, more severe microcephaly and the absence of motor milestones. Boys carrying mutations in *CDKL5* are much rarer and show more severe epileptic encephalopathy than girls [6, 7], probably due to the more severe consequences of dominant X-linked mutations in males than in females.

*CDKL5* encodes a ubiquitously expressed serine/threonine kinase whose catalytic domains share homology with members of the cyclin-dependent kinase family and mitogen-activated protein kinases [8]. CDKL5 is expressed at high levels in the brain, reaching a peak during postnatal development, when crucial events, such as neuronal maturation and synaptogenesis, occur [9, 10]. CDKL5 is mainly expressed in neurons and is distributed in the cell nucleus and cytoplasm, as it shuttles between these cellular compartments [9]. The identification of the molecular targets of CDKL5 has started to shed light on the role of CDKL5 in neurodevelopment. The CDKL5 substrates that have, so far, been identified are MeCP2 [11, 12], DNA methyltransferase 1 (DNMT1) [13], Rac1 [14], amphiphysin 1 [15], PSD-95 [16], NGL-1 [17], Mind Bomb 1 (Mib1) [18], Shootin1 [19], HDAC4 [20], and IQGAP1 [21]. Furthermore, different signaling networks are affected by the absence of CDKL5 [22]. Interestingly, loss of CDKL5 alters Akt-mTOR and Akt-GSK3-*β* signaling pathways [22–25]. Notably, mutations and dysfunction of components of these pathways have been implicated in the etiology of several neurodevelopmental disorders, such as autism, Rett syndrome, Fragile X syndrome, and tuberous sclerosis [26–28].

Several *Cdkl5* knockout (KO) mouse models have been generated to understand how CDKL5 dysfunction leads to neurological defects in CDKL5 disorder [22, 23, 29, 30]. Despite the lack of overt epilepsy, *Cdkl5* KO mice exhibit numerous behavioral deficits across motor, sensory, cognitive, and socioemotional domains that are reminiscent of human symptomatology [22–25, 29, 31, 32]. These behavioral defects are associated with morphological alterations, such as reduced dendritic arborization of hippocampal and cortical neurons, deficits in dendritic spine density and organization, and altered connectivity [20, 23–25, 33–35]. However, the majority of work involving *Cdkl5* KO mouse models has focused on the loss of Cdkl5 in male mice and only a few studies have included an initial characterization of heterozygous female *Cdkl5* KO mice [23, 25, 36]. An extensive behavioral characterization of heterozygous *Cdkl5* KO females is still missing, and this represents a critical gap, since heterozygous *Cdkl5* KO female mice better recapitulate the mosaic distribution of mutant cells present in the majority of CDKL5 patients. Given the clinical relevance of CDKL5 disorder in females, the aim of our current study was to evaluate the behavioral abnormalities in heterozygous *Cdkl5* KO female mice (*Cdkl5* +/−) compared to homozygous *Cdkl5* KO (*Cdkl5* −/−) and wild-type (*Cdkl5* +/+) female mice, focusing on cognitive (learning and memory) behaviors, motor activity and coordination, social behaviors, and breathing pattern, features that are particularly compromised in CDKL5 patients. Since behavioral abnormalities are the main biomarkers of CDKL5 disorder, our study may crucially define the validity of the heterozygous *Cdkl5* KO murine models for this disorder.

## 2. Experimental Procedures

### 2.1. Animal Husbandry

The mice used in this work derive from the *Cdkl5* null strain in the C57BL/6N background developed in [23] and backcrossed in C57BL/6J for three generations. Heterozygous *Cdkl5* KO (+/−) and homozygous *Cdkl5* KO (−/−) females were produced and karyotyped as previously described [23]. Age-matched controls (wild-type females (+/+), when possible littermates) were used for all experiments. Mice were housed three to five animals per cage and maintained on a 12 h light: 12 h dark cycle in a temperature- (23°C) and humidity-controlled environment with standard mouse chow and water ad libitum. The animals' health and comfort were controlled by the veterinary service. All research and animal care procedures were performed in accordance with the Italian and European Community law for the use of experimental animals and were approved by Bologna University Bioethical Committee. In this study, all efforts were made to minimize animal suffering and to keep the number of animals used to a minimum.

### 2.2. Western Blotting

Western blot analysis was conducted on independent cohorts of 3-week-, 8-week-, and 12–14-week-old mice (*n* = 3–8 for each genotype) that had not undergone behavioral assessment. Animals were euthanized with isoflurane (2% in pure oxygen) and sacrificed by cervical dislocation. The brain was quickly removed, and the somatosensory cortex, the hippocampal formation, and the cerebellum were homogenized in ice-cold RIPA buffer (50 mM Tris-HCl, pH 7.4, 150 mM NaCl, 1% Triton-X100, 0.5% sodium deoxycholate, and 0.1% SDS) supplemented with 1 mM PMSF and 1% protease and phosphatase inhibitor cocktail (Sigma-Aldrich). Extracts were immediately processed by Western blot or kept frozen (−80°C) until assayed. Protein concentration was determined by the Lowry method [37]. Equivalent amounts (10 *μ*g) of protein were subjected to electrophoresis on a BOLT Bis-Tris Plus gel (Thermo Fisher Scientific) and transferred to a Hybond ECL nitrocellulose membrane (Amersham Life Science). The following primary antibodies were used: sheep polyclonal anti-CDKL5 (1 : 1000, MRC Protein Phosphorylation and Ubiquitylation Unit—University of Dundee, UK), rabbit polyclonal anti-GAPDH (1 : 5000, Sigma-Aldrich), mouse monoclonal *β*-catenin (1 : 1000, BD Transduction Laboratories), rabbit polyclonal anti-phospho-AKT (Ser473) (1 : 1000), rabbit polyclonal anti-AKT (1 : 1000), rabbit polyclonal anti-phospho-ERK1/2 (1 : 1000), mouse monoclonal anti-ERK1/2 (1 : 1000), and rabbit polyclonal anti-phospho-MSK1 (Thr 581) (1 : 1000, Cell Signaling Technology). The following secondary antibodies were used: HRP-conjugated goat anti-sheep IgG, HRP-conjugated goat anti-mouse IgG, and HRP-conjugated goat anti-rabbit IgG (1 : 5000, Jackson ImmunoResearch) antibodies. Densitometric analysis of digitized images was performed using Chemidoc XRS Imaging Systems and Image Lab™ Software (Bio-Rad).

### 2.3. Histological and Immunohistochemistry Procedures

Histological analysis was conducted on independent cohorts of 14-week-old mice (*n* = 3 − 4 for each genotype) that had not undergone behavioral assessment. Animals were euthanized with isoflurane (2% in pure oxygen) and sacrificed by cervical dislocation. Brains were quickly removed and cut along the midline. Right hemispheres were processed for immunohistochemistry procedure, while left hemispheres were Golgi-stained as described below. All steps of sectioning, imaging, and data analysis were conducted blindly. *PSD-95 immunohistochemistry*—right hemispheres were fixed by immersion in 4% paraformaldehyde in 100 mM phosphate buffer, pH 7.4, stored in fixative for 48 h, kept in 20% sucrose for an additional 24 h, and then frozen with cold ice. Right hemispheres were cut with a freezing microtome into 30 *μ*m thick coronal sections that were serially collected. One out of 12 free-floating sections from the hippocampal formation were incubated for 24 h at 4°C with a rabbit polyclonal anti-PSD-95 antibody (1 : 1000; Abcam). Sections were then incubated at room temperature for 2 h with a CY3-conjugated goat anti-rabbit IgG (1 : 200; Jackson ImmunoResearch). For quantification of PSD-95 immunoreactive puncta, images from the molecular layer of the hippocampal dentate gyrus were acquired using a Leica TCS SL confocal microscope (Leica Mycrosytems; 63x oil immersion objective, NA 1.32; zoom factor = 8). Three to four sections per animal were analyzed. Counting was carried out using Image Pro Plus software (Media Cybernetics), and the number of PSD-95 immunoreactive puncta was expressed per *μ*m^2^. *Golgi staining*—left hemispheres were Golgi-stained using the FD Rapid GolgiStain TM Kit (FD NeuroTechnologies). Briefly, hemispheres were immersed in the impregnation solution containing mercuric chloride, potassium dichromate, and potassium chromate and stored at room temperature in darkness for 2–3 weeks. Hemispheres were cut with a microtome in 100 *μ*m thick coronal sections that were directly mounted on gelatin-coated slides and were air-dried at room temperature in the dark for an additional 2–3 days. After drying, sections were rinsed with distilled water and subsequently stained in the developing solution of the kit. A light microscope (Leica Mycrosystems) equipped with a motorized stage and focus control system and a color digital camera (CoolSNAP-Pro; Media Cybernetics) were used for neuronal tracing and to take bright-field images. Measurements were carried out using Image Pro Plus software (Media Cybernetics). *Neuronal tracing*—a series of sections across the whole rostrocaudal extent of the hippocampal dentate gyrus were used for reconstruction of Golgi-stained neurons. Golgi-stained neurons (15–20 per animal) were sampled from the outer part of the granule cell layer and traced with a dedicated software custom-designed for dendritic reconstruction (Immagini Computer, Milan, Italy), interfaced with Image Pro Plus (Media Cybernetics). The dendritic tree was traced live, at a final magnification of 500x, by focusing into the depth of the section. The operator starts with branches emerging from the cell soma and after having drawn the first parent branch goes on with all daughter branches of the next order in a centrifugal direction. At the end of tracing, the program reconstructs the total dendritic length, the mean length of branches, and the number of branches*. Spine density and morphology—*in Golgi-stained sections, spines of granule neurons were counted using a 100x oil immersion objective lens. Dendritic spine density was measured by manually counting the number of dendritic spines on dendritic segments in the molecular layer and expressed as the number of dendritic spines per *μ*m. Based on their morphology, dendritic spines can be divided into five different classes (immature spines: filopodium-like, thin-shaped, and stubby-shaped; mature spines: mushroom- and cup-shaped), which also reflect their state of maturation. The total number of spines was expressed per *μ*m, and the number of spines belonging to each class was counted and expressed as a percentage. About 200–250 spines from 25 to 30 dendrites derived from 12 to 15 neurons were analyzed per condition.

### 2.4. Behavioral Assays

All animal behavioral studies and analysis were performed blinded to genotype. Mice were allowed to habituate to the testing room for at least 1 h before the test, and testing was performed at the same time of day. All animal behaviors were tested at 12–16 weeks of age.

### 2.5. Test Cohorts

A total of 124 animals separated in six independent test cohorts were used for neurobehavioral studies. The sequence of the tests was arranged to minimize the effect of one test influencing subsequent evaluation of the next test, and mice were allowed to recover several days between different tests. The first test cohort consisted of 26 animals (+/+ *n* = 7, +/− *n* = 9, and −/− *n* = 10) that were tested with the following assays at indicated time points: buried food test, 12 weeks; accelerating rotarod assay, 13 weeks; open field, 14 weeks; and Morris water maze, 14–15 weeks. The second cohort consisted of 11 animals (+/+ *n* = 4, +/− *n* = 2, and −/− *n* = 5) that were tested with the following assays at indicated time points: buried food test, 12 weeks; open field, 14 weeks; and Morris water maze, 14–15 weeks. The third cohort consisted of 19 animals (+/+ *n* = 5, +/− *n* = 8, and −/− *n* = 7) that were tested with the following assays at the indicated time points: nesting, 12 weeks; accelerating rotarod assay, 12 weeks; marble burying, 13 weeks; open field, 14 weeks; Morris water maze, 14–15 weeks; and passive avoidance, 16 weeks. The fourth test cohort consisted of 19 animals (+/+ *n* = 8, +/− *n* = 4, and −/− *n* = 7) that were tested with the following assays at the indicated time points: accelerating rotarod assay, 12 weeks; marble burying, 13 weeks; Morris water maze, 14–15 weeks; and passive avoidance, 16 weeks. The fifth test cohort consisted of 23 animals (+/+ *n* = 5, +/− *n* = 8, and −/− *n* = 10) that were tested for nesting at 12 weeks of age or with the buried food test or the marble burying test at 13 weeks of age. The sixth test of cohort consisted of 26 animals (+/+ *n* = 9, +/− *n* = 11, and −/− *n* = 8) that were tested for breathing abnormalities at 14 weeks and body weight at 8 and 14 weeks.

### 2.6. Buried Food Test

The buried food test relies on the animal's ability to smell volatile odors and its natural tendency to use olfactory cues for foraging. The testing protocol of 3 days consists of an odor familiarization exercise on day 1, food deprivation on day 2, and testing on day 3. On day 1, mice were placed in a clean cage and a piece of cookie (exactly the same kind as in the test) was placed in each cage and left overnight. Cages were inspected on day 2 to verify that the cookies had been consumed, thus verifying that the bait was highly palatable. On day 2, mice were deprived of food for 24 h, and the test was performed on day 3. Mice were individually introduced into a clean cage containing 4 cm of deep clean bedding and allowed to acclimate to the cage for 5 min to reduce the interference of novel environment exploration during the test. A cookie was buried beneath 1 cm of bedding in a random corner of the cage, and the mouse was introduced into the cage. The time necessary for the animal to retrieve the cookie with its front paws was measured in seconds (latency) up to a maximum of 15 min (900 s).

### 2.7. Nesting

Nest building ability was evaluated as proposed by Deacon [38]. Animals were placed in individual cages with standard bedding, and a standard piece of paper towel (23 cm × 23 cm) was provided. The nests were independently assessed at 3 and 22 h by two operators using the following scoring system: 0—no nest, 1—primitive flat nest (pad-shaped, consisting of a flat paper tissue which slightly elevates a mouse above the bedding), 2—more complex nest (including warping and biting the paper towel), 3—complex accurate cup-shaped nests (with shredded paper interwoven to form the walls of the cup), and 4—complex hooded nest, with walls forming a ceiling so the nest becomes a hollow sphere with one opening.

### 2.8. Marble Burying

The marble burying test was performed by placing animals individually in a home-cage-like environment with 5 cm of unscented standard bedding material and 20 marbles (14.3 mm in diameter) arranged in a 4 × 5 matrix and left undisturbed for 30 min. The number of marbles that were at least two-thirds buried at the end of the trial was counted.

### 2.9. Accelerating Rotarod Assay

Before the first test session, animals were briefly trained at a constant speed of 5 rpm on the rotarod apparatus (Ugo Basile) for 30 s. Thirty minutes later, testing was performed at an accelerating linear speed (5–35 rpm within 270 s + 30 s max speed). Four testing trials with an intertrial interval of 1 h were performed. The latency to fall from the rotating rod and the number of passive rotations (rotation in which the mouse does not perform any coordinated movement but is passively transported by the rotating apparatus) were recorded for each trial.

### 2.10. Open Field

In order to assess locomotion, animals were placed in the center of a square arena (50 × 50 cm) and their behavior was monitored for 20 min using a video camera placed above the center of the arena. Distinct features of locomotor activity, including total distance traveled, average locomotion velocity, and the time spent at the border, by the walls, and in the center, were scored by EthoVision10XT software (Noldus Information Technology B.V., The Netherlands). The numbers of stereotypical jumps (repetitive beam breaks < 1 s) in the corners of the arena were manually counted by a trained observer who was blind to the genotype. The test chambers were cleaned with 70% ethanol between test subjects. To evaluate whether *Cdkl5* KO female mice show stereotypic behaviors also in the home-cage environment, animals were single-caged for 48 h and stereotypic jumps were evaluated in a time window of 20 min.

### 2.11. Morris Water Maze

Hippocampal-dependent spatial learning and memory was assessed using the Morris water maze (MWM). Mice were trained to locate a hidden escape platform in a circular pool. The apparatus consisted of a circular water tank (1 m in diameter, 50 cm high) with a transparent round escape platform (10 cm^2^) placed in a fixed position. The tank was filled with tap water at a temperature of 22°C up to 0.5 cm above the top of the platform, and the water was made opaque with milk. In the experimental room, intramaze and extramaze visual cues were placed to enable spatial orientation. Mouse behavior was automatically videotracked (EthoVision 3.1; Noldus Information Technology B.V.). During training, each mouse was subjected to either 1 swimming session of 4 trials (day 1) or 2 sessions of 4 trials per day (days 2–5), with an intersession interval of 1 h (acquisition phase). Mice were allowed to search for the platform for up to 60 s. If a mouse did not find the platform, it was gently guided to it and allowed to remain there for 15 s. During the intertrial-interval (15 s), mice were placed in an empty cage. The latency to find the hidden platform was used as a measure of learning. Twenty-four h after the last acquisition trial, on day 6, the platform was removed and a probe test was run. Animals were allowed to search for the platform for up to 60 s. The latency of the first entrance into the former platform area, the frequency of entrances and the time spent in the quadrant in which the platform had been located during training, and the proximity to the former platform quadrant (Gallagher's test) were employed as measures of retention of acquired spatial preference. During the learning phase, the average and maximum swim speeds were also analyzed. The percentage of floating was defined as the percentage of time swimming at a speed slower than 4 cm/s. Animals that failed the visual clue test were excluded from the analysis (+/− *n* = 1 and −/− *n* = 3).

### 2.12. Passive Avoidance

For the passive avoidance task, a memory task that involves contributions from both the hippocampus and amygdala, we used a tilting-floor box (47 × 18 × 26 cm) divided into 2 compartments (lit and dark) by a sliding door and a control unit incorporating a shocker (Ugo Basile). This classic instrument for Pavlovian conditioning exploits the tendency in mice to escape from an illuminated area into a dark one (step-through method). In general, mice step quickly through the gate and enter the dark compartment (innate preference for the dark compartment). Upon entering the dark compartment, mice received a brief mild foot shock (0.4 mA for 3 s) and were removed from the chamber after a 15 s delay. The chambers were cleaned with 70% ethanol between testing one subject and another. After a 24 h retention period, mice were placed back into the illuminated compartment and the latency to reenter the dark chamber was measured up to 358 s.

### 2.13. Noninvasive Assessment of Sleep and Breathing Pattern

Hypnic and respiratory phenotypes of mice were assessed noninvasively with a validated technique based on whole-body plethysmography (WBP) [39, 40]. Briefly, each mouse was placed inside a WBP chamber flushed with air at 1.5 l/h for the first 8 h of the light period. The respiratory (WBP chamber pressure) signal was continuously recorded together with chamber humidity and temperature, digitized, and stored at 128 Hz, 4 Hz, and 4 Hz, respectively. The system was calibrated with a 100 *μ*l micro-syringe (Hamilton, Reno, USA) at the end of each recording. The states of wakefulness, nonrapid eye movement sleep (NREMS), and rapid eye movement sleep (REMS) were scored based on inspection of the raw WBP signal with the investigators blinded to the animal's genotype. Quantitative analysis of breathing was restricted to stable sleep episodes ≥ 12 s because of the frequent occurrence of movement artefacts during wakefulness. Apneas were automatically detected as breaths with instantaneous total breath duration (T_TOT_) > 3 times; the average T_TOT_ for each mouse and sleep state and detection accuracy were checked on raw recordings.

### 2.14. Statistical Analysis

Data from single animals represented the unity of analysis. Results are presented as mean ± standard error of the mean (± SE). Statistical analysis was performed with SPSS (version 23) or GraphPad Prism (version 6). All datasets were analyzed using the ROUT method (*Q* = 1%) to identify significant outliers and the Shapiro-Wilk test for normality. Datasets with normal distribution were analyzed for significance using a one-way analysis of variance (ANOVA) with genotype as a factor. Post hoc multiple comparisons were carried out using the Fisher least significant difference (Fisher's LSD) test. Datasets with nonparametric distribution were analyzed using the Kruskal-Wallis test. Post hoc multiple comparisons were carried out using Dunn's multiple comparison test or the Mann–Whitney test. For the rotarod assay and the learning phase of the MWM test, statistical analysis was performed using a repeated ANOVA. For categorical data, that is, percentages of spines, we used a chi-squared test. A probability level of *p* < 0.05 was considered to be statistically significant. Data from several neurobehavioral assays were analyzed with the receiver operating characteristic (ROC) functions and the relative area under the curve (AUC), and correspondent *p* values are reported in Supplementary Table 1. This analysis enables the assessment of the ability of a test to discriminate between control and diseased animals. The area under the curve (AUC) reflects the specificity and sensitivity of a test and ranges from totally noninformative (AUC = 0.5) to perfect test (AUC = 1) [41, 42].

## 3. Results

### 3.1. Cdkl5 Expression Levels in the Brain of *Cdkl5* +/− and *Cdkl5* −/− Female Mice

**Figure 1:**
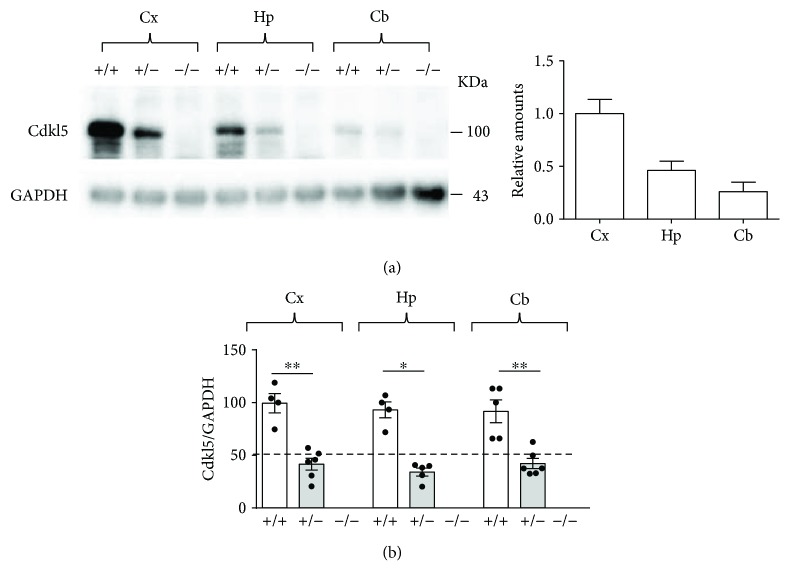
Intermediate Cdkl5 expression levels in the brain of *Cdkl5* +/− female mice. (a, b) Western blot analysis of *Cdkl5* levels normalized to GAPDH (glyceraldehyde 3-phosphate dehydrogenase) levels in the cortex (Cx), hippocampus (Hp), and cerebellum (Cb) of wild-type (*Cdkl5* +/+ *n* = 5), heterozygous (*Cdkl5* +/− *n* = 6), and homozygous (*Cdkl5* −/− *n* = 5) *Cdkl5* KO female mice aged 3–4 months. Immunoblots in (a) are examples from one animal of each experimental group, and the graph shows the relative amounts of Cdkl5 expression in the different brain structures of *Cdkl5* +/+ mice. Regardless of the relative amounts, Cdkl5 expression levels were reduced to about 35–46% in *Cdkl5* +/− mice in comparison to wild-type mice and absent in *Cdkl5* −/− mice in all analyzed brain regions (b). Data in (b) are expressed as a percentage of the values of *Cdkl5* +/+ mice. Values are represented as means ± SE. ^∗^ *p* < 0.05 and ^∗∗^ *p* < 0.001 (Mann–Whitney test after unpaired *t*-test).

To determine the degree of Cdkl5 levels in the brain of female *Cdkl5* KO mice, we performed Western blot assays on total protein extracts from various brain regions from adult (3–4 months of age) wild-type (*Cdkl5* +/+), heterozygous (*Cdkl5* +/−), and homozygous (*Cdkl5* −/−) *Cdkl5* KO female mice. The Cdkl5 signal was normalized to GAPDH (glyceraldehyde 3-phosphate dehydrogenase) levels, and the relative amounts are illustrated in Figure 1(a). Western blot analyses showed that Cdkl5 expression levels were not uniform among the cortex, hippocampus, and cerebellum (Figure 1(a)). In fact, Cdkl5 levels were higher in the cortex, compared with the hippocampus and cerebellum (Figure 1(a)), while the cerebellum was the brain region that showed the lowest Cdkl5 expression levels (Figure 1(a)). Regardless of the relative amounts, in all analyzed brain regions Cdkl5 expression levels were reduced to about 35–46% in *Cdkl5* +/− mice in comparison to wild-type mice (Mann–Whitney test: Cx: *U* = 0, *p* = 0.0095; Hp: *U* = 0, *p* = 0.0159; Cb: *U* = 0, *p* = 0.0043) and were absent in *Cdkl5* −/− mice (Figure 1(b)).

### 3.2. Reduced Body Weight and Olfactory Performance in *Cdkl5* +/− and *Cdkl5* −/− Female Mice

**Figure 2:**
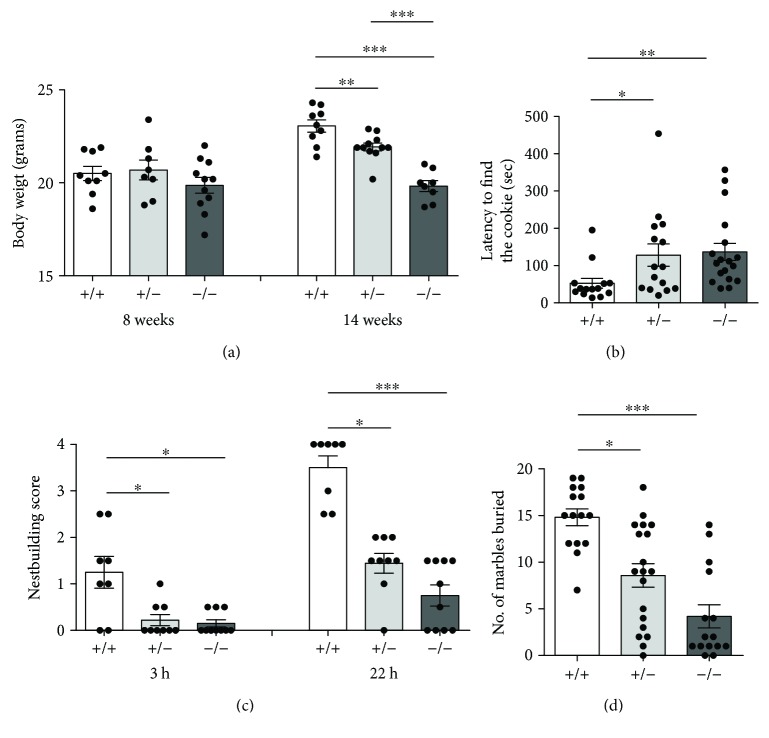
Reduced body weight, impaired olfaction, and autistic-like features in *Cdkl5* +/− female mice. (a) Body weight at 8 and 14 weeks of age in *Cdkl5* +/+ (*n* = 9), *Cdkl5* +/− (*n* = 8 and *n* = 11, resp.), and *Cdkl5* −/− (*n* = 11 and *n* = 8, resp.) mice. Only at 14 weeks of age *Cdkl5* +/− and *Cdkl5* −/− mice showed reduced body weight compared to *Cdkl5* +/+ mice. (b) Olfactory ability evaluated using the buried food test. *Cdkl5* +/− (*n* = 15) and *Cdkl5* −/− (*n* = 18) mice showed an increased latency to find the buried cookie compared to *Cdkl5* +/+ mice (*n* = 14), indicating olfactory impairment in *Cdkl5* +/− and *Cdkl5* −/− mice. (c) Nest building ability evaluated at 3 and 22 h. *Cdkl5* +/− (*n* = 9) and *Cdkl5* −/− (*n* = 10) mice showed impaired nesting behavior compared to *Cdkl5* +/+ (*n* = 8) mice. (d) Marble burying test. *Cdkl5* +/− (*n* = 19) and *Cdkl5* −/− (*n* = 15) mice buried fewer marbles compared to *Cdkl5* +/+ mice (*n* = 15). Values represent mean ± SEM. ^∗^ *p* < 0.05, ^∗∗^ *p* < 0.01, and ^∗∗∗^ *p* < 0.001 (datasets in (a), Fisher's LSD test after ANOVA; datasets in (b–d), Dunn's test after Kruskal-Wallis).

We first evaluated whether mutant *Cdkl5* female mice showed a significant difference in body weight. As previously reported [23], no significant weight differences were observed in young adult female *Cdkl5* KO mice [*F*_(2, 25)_ = 0.9982, *p* = 0.3828] (8 weeks old; Figure 2(a)). However, at 14 weeks of age, the average weight of *Cdkl5* +/− and *Cdkl5* −/− female mice was lower compared with wild-type (*Cdkl5* +/+) females [*F*_(2, 25)_ = 31.66, *p* < 0.0001; Fisher's LSD: *p* = 0.0065 and *p* < 0.0001, resp.]. *Cdkl*5 +/− females achieved an intermediate body weight between that of *Cdkl5* +/+ and *Cdkl5* −/− female mice.

Among the many sensory stimuli that influence behavioral decisions regarding food choice, olfactory inputs are likely to contribute to food uptake and the regulation of energy homeostasis [43]. Using the buried food test, we evaluated the ability of *Cdkl5* +/− and *Cdkl5* −/− female mice to smell volatile odors. We found that 3 month old *Cdkl5* +/− and *Cdkl5* −/− female mice showed a reduced olfactory performance compared with wild-type (*Cdkl5* +/+) females [Kruskal-Wallis, *p* = 0.0019; Dunn's test: *p* = 0.0387 and *p* = 0.0016, resp.] (Figure 2(b)), suggesting an olfactory impairment in the *Cdkl5* KO mice.

All the behavioral experiments described below were performed on female *Cdkl5* KO mice at 3–4 months of age.

### 3.3. Autistic-Like Features in *Cdkl5* +/− and *Cdkl5* −/− Female Mice

To investigate whether loss of Cdkl5 function in *Cdkl5* +/− and *Cdkl5* −/− female mice is associated with autistic-like (ASD-like) phenotypes, we first analyzed home-cage social behaviors (nest building ability and marble burying). *Cdkl5* +/− and *Cdkl5* −/− displayed impaired nesting compared to control mice. After 3 h, *Cdkl5* +/+ female mice showed initial structured nests with a median score of 1, while *Cdkl5* +/− and *Cdkl5* −/− mice showed a significantly lower median nest quality with most of the mice leaving the material untouched [Kruskal-Wallis, *p* = 0.0133; Dunn's test: *p* = 0.0461 and *p* = 0.0202, resp.] (Figure 2(c)). Even after 22 h, the majority of *Cdkl5* +/− and *Cdkl5* −/− mice failed to build complex structured nests with a median nest quality of 1.4 and 0.8, respectively, in contrast to *Cdkl5* +/+ mice who had a median nest quality of 3.5 [Kruskal-Wallis, *p* < 0.0001; Dunn's test: *p* = 0.0131 and *p* < 0.0001, resp.] (Figure 2(c)).

The marble burying test was used to evaluate exploration and environmental interest. Since marble burying may depend not only on interest in the environment but also on stress novelty, animals were tested after 5 min of habituation. *Cdkl5* +/− and *Cdkl5* −/− mice buried a significantly lower number of marbles compared to *Cdkl5* +/+ mice [Kruskal-Wallis, *p* < 0.0001; Dunn's test: *p* = 0.0110 and *p* < 0.0001, resp.] (Figure 2(d)), suggesting that *Cdkl5* +/− and *Cdkl5* −/− female mice had a decreased interest in the environment.

### 3.4. Impaired Motor Function in *Cdkl5* +/− and *Cdkl5* −/− Female Mice

**Figure 3:**
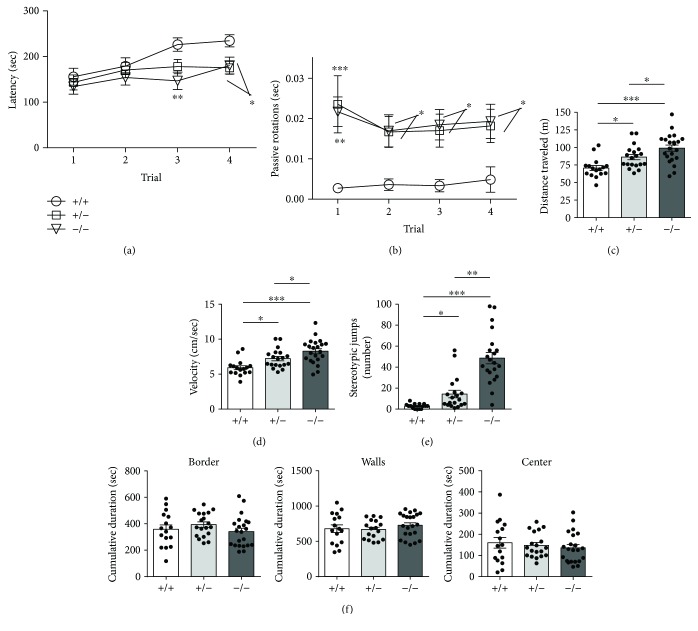
Impaired motor coordination, hyperactivity, and stereotypes in *Cdkl5* +/− female mice. (a, b) Rotarod assay, measuring latency to fall (a) and frequency of passive rotations (b; rotations in which the mouse does not perform any coordinated movement but is passively transported from the rotating apparatus) on the accelerating rotating rod. Testing was performed in 4 trials with an intertrial interval of 1 h. *Cdkl5* +/− (*n* = 21) and *Cdkl5* −/− (*n* = 23) mice showed a decreased latency to fall (a) and an increased frequency of passive rotations compared to *Cdkl5* +/+ (*n* = 17) mice, indicating impaired motor coordination in *Cdkl5* +/− and *Cdkl5* −/− mice. (c, d) Locomotor activity measured as total distance traveled (c) and average locomotion velocity (d) during a 20 min open-field test. *Cdkl5* +/− (*n* = 19) and *Cdkl5* −/− (*n* = 22) mice exhibited increased locomotor activity with a longer total distance traveled (c) at a greater average speed (d) compared to *Cdkl5* +/+ (*n* = 16) mice. (e) Number of stereotypic jumps (repetitive beam breaks < 1 s) in the corners of the open-field arena during the 20 min trial. *Cdkl5* +/− (*n* = 19) and *Cdkl5* −/− (*n* = 21) mice showed an increased number of repetitive stereotyped jumps compared to *Cdkl5* +/+ (*n* = 15) mice. (f) Time (cumulative duration) spent by the border, near the walls, and in the center of the open-field arena. *Cdkl5* +/+, *Cdkl5* +/−, and *Cdkl5* −/− mice spent a comparable time at the border, near the walls, and in the center compared to controls, suggesting that hyperactivity was not due to increased anxiety. Values represent mean ± SEM. ^∗^ *p* < 0.05, ^∗∗^ *p* < 0.01, and ^∗∗∗^ *p* < 0.001 (datasets in (a–d, f), Fisher's LSD test after ANOVA; datasets in (e) Dunn's test after Kruskall-Wallis).

To assess whether motor impairment is present in female *Cdkl5* KO mice, we assessed motor function of *Cdkl5* +/− and *Cdkl5* −/− female mice on an accelerating rotarod assay and in the open-field test. Mice were tested on a rotating rod for 4 trials with an intertrial interval of 1 h, and the latency to fall from the rotating rod was evaluated. *Cdkl5* −/− exhibited significantly reduced falling latency compared to *Cdkl5* +/+ mice in the third and fourth trials [*F*_(2, 58)_ = 3.591, *p* = 0.0339; Fisher's LSD: *p* = 0.0013 and *p* = 0.0274, resp.] (Figure 3(a)). Similarly, in *Cdkl5* +/− mice the latency to fall was reduced in the last two trials (Figure 3(a)), although the difference was statistically significant only in the fourth trial [Fisher's LSD: trial 3: *p* = 0.053, trial 4: *p* = 0.0185], suggesting that loss of CDKL5 impairs not only motor coordination but also motor learning skills. We further evaluated the frequency of passive rotations (number of passive rotations/sec), rotations in which the mouse does not perform any coordinated movement and is passively transported from the rotating apparatus. Rotarod performance notably differed among groups, with *Cdkl5* +/− and *Cdkl5* −/− mice showing significantly more passive rotations during the four trials [*F*_(2, 58)_ = 11.94, *p* < 0.0001; Fisher's LSD: trial 1: *p* = 0.0006 and *p* = 0.0013; trial 2: *p* = 0.0291 and *p* = 0.0234; trial 3: *p* = 0.0225 and *p* = 0.0102; trial 4: *p* = 0.0262 and *p* = 0.0139, resp.] (Figure 3(b)), confirming that both *Cdkl5* +/− and *Cdkl5* −/− female mice exhibit impaired motor coordination.

To further evaluate locomotor activity, we performed an open-field test. *Cdkl5* +/− and *Cdkl5* −/− mice traveled a significantly longer distance [*F*_(2, 54)_ = 11.90, *p* < 0.0001; Fisher's LSD: *p* = 0.0116 and *p* < 0.0001, resp.] (Figure 3(c)) and moved at a higher average speed [*F*_(2, 54)_ = 11.89, *p* < 0.0001; Fisher's LSD: *p* = 0.0116 and *p* < 0.0001, resp.] (Figure 3(d)) compared to their controls (*Cdkl5* +/+), indicating elevated locomotor activity in *Cdkl5* mutants. To determine whether this hyperactivity was caused by increased anxiety, we compared the time spent in the border area, near the walls, and in the center of the arena (Figure 3(f)). *Cdkl5* +/+, *Cdkl5* +/−, and *Cdkl5* −/− spent a comparable time at the border [*F*_(2, 54)_ = 1.135, *p* = 0.3289], near the walls [*F*_(2, 54)_ = 0.6457, *p* = 0.5285], and in the center [*F*_(2, 54)_ = 0.4563, *p* = 0.6361] (Figure 3(f)), suggesting that the increased locomotor activity is not due to increased anxiety.

In addition to motor dysfunction, stereotypic movements frequently cooccur in CDKL5 patients [3]. Therefore, we evaluated stereotypic behavior in *Cdkl5* +/− and *Cdkl5* −/− mice by counting the number of repetitive jumps in the corners of the open-field arena. *Cdkl5* +/− and *Cdkl5* −/− showed an increased number of stereotypical jumps compared to wild-type females [Kruskal-Wallis, *p* < 0.0001; Dunn's test: *p* = 0.0362 and *p* < 0.0001, resp.] (Figure 3(e)). An increased number in vertical jumps were also observed in *Cdkl5* KO female mice in the home-cage environment (*Cdkl5* +/+ 0 ± 0, *Cdkl5* +/− 2.2 ± 1.9, and *Cdkl5* −/− 1.8 ± 1.8), suggesting that the stereotypic behavior was not caused by the stressful situation (the new environment).

### 3.5. Impaired Learning and Memory Performance in *Cdkl5* +/− and *Cdkl5* −/− Female Mice

**Figure 4:**
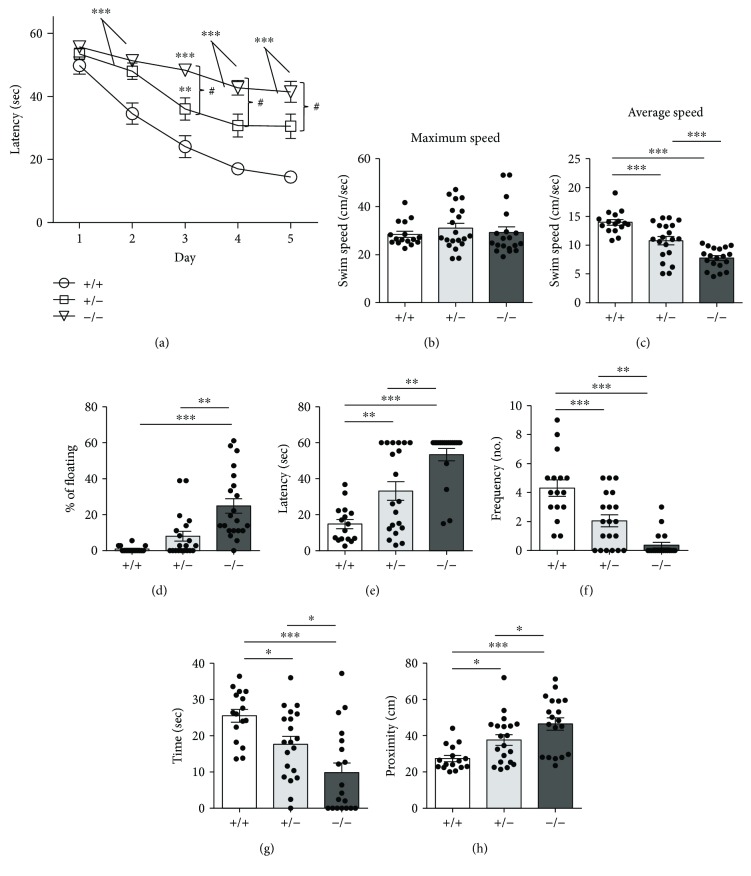
Impaired hippocampus-dependent learning and memory in *Cdkl5* +/− female mice. (a) Spatial learning assessed using the Morris water maze. *Cdkl5* +/− (*n* = 20) and *Cdkl5* −/− (*n* = 19) mice showed an increased latency to find the platform over the 5-day learning period compared to the *Cdkl5* +/+ (*n* = 16) mice. (b, c) Maximum (b) and average (c) swim speed during the learning phase in mice as in (a). (d) Percentage of floating, defined as the percentage of time swimming at less than 4 cm/s during the learning phase in mice as in (a). (e–h) On day 6 (probe test), the platform was removed and spatial memory was assessed by evaluating different parameters. *Cdkl5* +/− and *Cdkl5* −/− mice showed an increased latency to enter the former platform zone (e), a reduced frequency to enter (f), and time spent in the quadrant (g) in which the platform had been located during the learning period. In addition, *Cdkl5* +/− and *Cdkl5* −/− mice swam a larger distance from the former platform (h) compared to the *Cdkl5* +/+ mice, indicating defects in spatial memory. Values represent mean ± SEM. ^∗^ *p* < 0.05, ^∗∗^ *p* < 0.01, and ^∗∗∗^ *p* < 0.001 as compared to the *Cdkl5* +/+ female mice; ^#^ *p* < 0.05 as compared to the *Cdkl5* +/− female mice (dataset in a); ^∗^ *p* < 0.05, ^∗∗^ *p* < 0.01, and ^∗∗∗^ *p* < 0.001 as compared to the *Cdkl5* +/+ or Cdkl5 +/− female mice (dataset in b–h), Fisher's LSD test after ANOVA; datasets in (d), Dunn's test after Kruskall-Wallis).

To examine whether *Cdkl5* +/− and *Cdkl5* −/− female mice show cognitive impairment, hippocampus-dependent learning and memory were evaluated using the Morris water maze (MWM). Mice were tested for their ability to find a hidden platform for 5 days (learning phase) and were subjected to the probe test on day 6. A repeated ANOVA on escape latency during the learning phase revealed a significant genotype effect [*F*_(2, 52)_ = 28.03, *p* < 0.0001]. In the learning phase, both *Cdkl5* +/− and *Cdkl5* −/− mice showed a reduced ability to learn over time compared to *Cdkl5* +/+ mice [Fisher's LSD: day 2: *p* = 0.0010 and *p* < 0.0001; day 3: *p* = 0.0033 and *p* < 0.0001; day 4: *p* = 0.0008 and *p* < 0.0001; day 5: *p* < 0.0001 and *p* < 0.0001, resp.] (Figure 4(a)). While *Cdkl5* +/+ mice exhibited a fast learning improvement with time and learned to find the platform by the second day [Fisher's LSD: *p* < 0.0001], significant learning was detected in *Cdkl5* +/− and *Cdkl5 −/−* mice by the third day [Fisher's LSD: *p* < 0.0001 and *p* = 0.015, resp.], indicating a learning deficit in both genotypes. No significant differences in the learning curves were observed between the *Cdkl5* +/− and *Cdkl5* −/− mice in the first two days of training (Figure 4(a)), while *Cdkl5* −/− mice showed a greater impairment in finding the hidden platform in the following days [Fisher's LSD: day 3: *p* = 0.0015; day 4: *p* = 0.0020; and day 5: *p* = 0.0049].

Performance in the MWM is influenced by sensorimotor function and motivation, and these parameters can be assessed using swimming patterns such as swim speed and floating (rest time). No difference in maximum swim speed was observed among *Cdkl5* +/+, *Cdkl5* +/−, and *Cdkl5* −/− mice [*F*_(2, 52)_ = 0.4431, *p* = 0.6445] (Figure 4(b)), indicating that the deficit of *Cdkl5* +/− and −/− mice in the hidden platform test was not caused by abnormalities in locomotor activity or coordination. On the contrary, average swim speed significantly decreased in *Cdkl5* +/− mice and *Cdkl5* −/− mice [*F*_(2, 52)_ = 28.21, *p* < 0.0001; Fisher's LSD: *p* = 0.0003 and *p* < 0.0001, resp.] (Figure 4(c)). Even relatively short intervals of floating behavior can cause significant changes in the mean raw average speed of a trial. We quantified the number of trials with floating episodes during training across genotypes (Figure 4(d)). An analysis of floating episodes revealed that *Cdkl5* −/− mice showed a significantly higher percentage of floating behaviors compared to *Cdkl5* +/+ mice [Kruskal-Wallis, *p* < 0.0001; Dunn's test: *p* < 0.0001] (Figure 4(d)), while *Cdkl5* +/− mice showed a slightly, but not significantly, increased number of floating episodes [Dunn's test: *p* = 0.1716] (Figure 4(d)). These results suggest that an increased level of floating behavior can account for the reduced swimming speed of *Cdkl5* +/− and *Cdkl5* −/− mice. Thigmotaxis (swimming along the walls of the pool) was not observed among *Cdkl5* female genotypes.

In the probe test, the following parameters were considered as an index of spatial memory: (i) latency to enter the former platform zone (Figure 4(e)), (ii) frequency of entrances into the former platform quadrant (Figure 4(f)), (iii) time spent in the former platform quadrant (Figure 4(g)), and (iv) proximity to the former platform position (Gallagher's test) (Figure 4(h)). Performance in all examined parameters was significantly impaired in *Cdkl5* +/− and *Cdkl5* −/− mice. Both showed an increase in the latency to enter the former platform zone [*F*_(2, 52)_ = 21.70, *p* < 0.0001; Fisher's LSD: *p* = 0.0026 and *p* < 0.0001, resp.] (Figure 4(e)), a reduction in the frequency of entrances [*F*_(2, 52)_ = 22.72, *p* < 0.0001; Fisher's LSD: *p* = 0.0003 and *p* < 0.0001, resp.] (Figure 4(f)), a reduction in the time spent there [*F*_(2, 52)_ = 11.33, *p* < 0.0001; Fisher's LSD: *p* = 0.0195 and *p* < 0.0001, resp.] (Figure 4(g)), and a reduction in the proximity to the platform quadrant [*F*_(2, 52)_ = 10.25, *p* = 0.0002; Fisher's LSD: *p* = 0.0176 and *p* < 0.0001, resp.] (Figure 4(h)).

**Figure 5:**
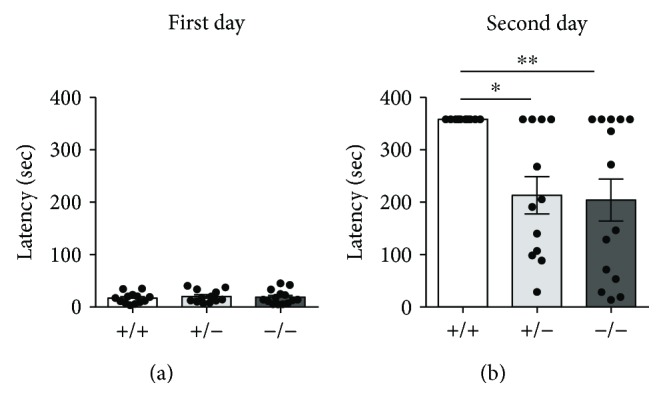
Impaired memory in *Cdkl5* +/− female mice. (a, b) Passive avoidance task to measure memory that involves contributions from both the hippocampus and amygdala. On the first day (a), no difference was observed between genotypes regarding the latency to enter the dark compartment. On the second day, *Cdkl5* +/− (*n* = 12) and *Cdkl5* −/− (*n* = 14) mice showed a decreased latency to enter the dark compartment compared to *Cdkl5* +/+ (*n* = 13) mice, indicating defects in memory. Values represent mean ± SEM. ^∗^ *p* < 0.05 and ^∗∗^ *p* < 0.01 (Dunn's test after Kruskall-Wallis).

To confirm the observed defects in memory, we performed a second test, the passive avoidance test. In this task, animals are conditioned with a single aversive event (mild foot shock) and 24 h later are tested for recollection of that experience. Figures 5(a) and 5(b) show the latency to enter the dark compartment on the first and second days of the test. While no difference in step-through latency was observed on the first day [Kruskal-Wallis, *p* = 0.8028] (Figure 5(a)), *Cdkl5* +/− and *Cdkl5* −/− mice show a significantly decreased latency on the second day [Kruskal Wallis, *p* = 0.0049; Dunn's test: *p* = 0.0209 and *p* = 0.0073, resp.] (Figure 5(b)), suggesting a defect in associative memory.

### 3.6. Abnormal Breathing Pattern in *Cdkl5* +/− and *Cdkl5* −/− Female Mice

**Figure 6:**
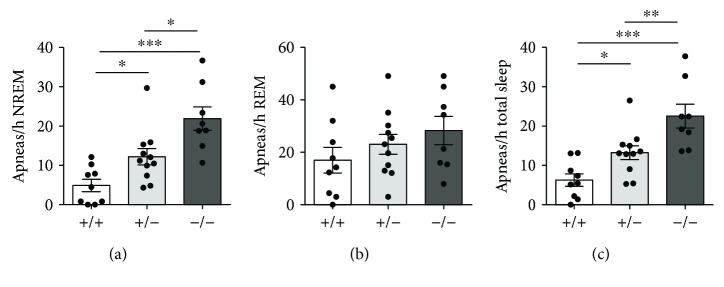
Sleep apnea occurrence rate in *Cdkl5* +/− female mice. (a, c) Apnea occurrence rate during NREM sleep (a), REM sleep (b), and total sleep period (c). *Cdkl5* +/− (*n* = 11) and *Cdkl5* −/− (*n* = 8) mice showed an increased number of apneas during NREM sleep (a) and, consequently, an increased number of total sleep apneas (c) compared to *Cdkl5* +/+ (*n* = 9) mice. Values represent mean ± SEM. ^∗^ *p* < 0.05, ^∗∗^ *p* < 0.01, and ^∗∗∗^ *p* < 0.001 (Fisher's LSD test after ANOVA).

To determine whether breathing irregularities and sleep disturbance are present in *Cdkl5* +/− and *Cdkl5* −/− females, hypnic and respiratory phenotypes were assessed using noninvasive whole-body plethysmography (WBP). *Cdkl5* +/− and *Cdkl5* −/− females had more frequent apneas during NREM sleep [*F*_(2, 25)_ = 13.26, *p* = 0.0001; Fisher's LSD: *p* = 0.0249 and *p* < 0.0001, resp.] and during total sleep time [*F*_(2, 25)_ = 13.57, *p* = 0.0001; Fisher's LSD: *p* = 0.0238 and *p* < 0.0001, resp.] compared to *Cdkl5* +/+ female mice (Figures 6(a) and 6(c)), but not during REM sleep [*F*_(2, 25)_ = 1.384, *p* = 0.2691] (Figure 6(b)), confirming the presence of an impaired breathing pattern in heterozygous and homozygous *Cdkl5* female mice.

### 3.7. *Cdkl5* +/− and *Cdkl5* −/− Female Mice Exhibit Altered Neuronal Maturation and Synapse Development

**Figure 7:**
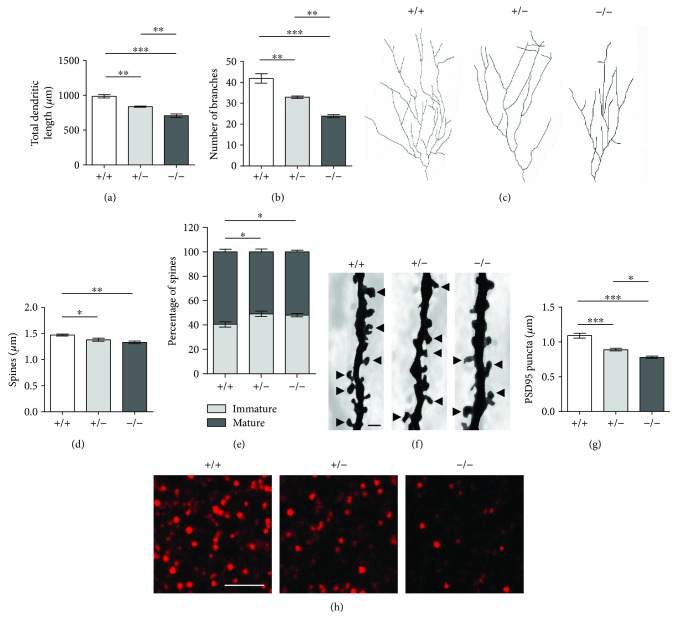
Impaired hippocampal development in *Cdkl5* +/− female mice. (a, b) Mean total dendritic length (a) and mean number of dendritic branches (b) of Golgi-stained mature granule neurons of *Cdkl5* +/+ (*n* = 4), *Cdkl5* +/− (*n* = 3), and *Cdkl5* −/− (*n* = 4) mice. (c) Examples of the reconstructed dendritic tree of Golgi-stained mature granule neurons of one animal from each experimental group. (d, e) Dendritic spine density (number of spines per μm; d) and percentage of immature and mature spines (e) in relation to the total number of protrusions of granule neurons of mice as in (a). (f) Examples of Golgi-stained dendritic branches of mature granule neurons of one animal from each experimental group. The black triangles indicate mature spines (mushroom- and cup-shaped spines). Scale bar: 1 *μ*m. (g) Number of fluorescent puncta per *μ*m^2^ exhibiting PSD-95 immunoreactivity in the molecular layer of the dentate gyrus of *Cdkl5* +/+ (*n* = 4), *Cdkl5* +/− (*n* = 4), and *Cdkl5* −/− (*n* = 3) mice. (h) Confocal images of sections processed for PSD-95 immunohistochemistry of one animal from each experimental group. Scale bar: 2.5 *μ*m. Values represent mean ± SEM. ^∗^ *p* < 0.05, ^∗∗^ *p* < 0.01, and ^∗∗∗^ *p* < 0.001 (datasets in (a–c, e–h), Fisher's LSD test after ANOVA; datasets in (d), Dunn's test after Kruskall-Wallis).

Changes in neuronal morphology, including defects in neuronal maturation, alterations in the organization and stability of dendritic spines, and defects in synaptogenesis, have been consistently reported in male *Cdkl5* KO mice [20, 22–25, 33–35]. To determine whether similar defects were also present in heterozygous and homozygous *Cdkl5* female mice, we analyzed dendritic development and spine density/morphology in Golgi-stained granule neurons of the dentate gyrus (DG) from 3–4 month-old females. Total dendritic length [*F*_(2, 8)_ = 40.28, *p* < 0.0001; Fisher's LSD: *p* = 0.0023 and *p* < 0.0001, resp.] and number of dendritic branches [*F*_(2, 8)_ = 37.34, *p* < 0.0001; Fisher's LSD: *p* = 0.0041 and *p* < 0.0001, resp.] of granule neurons were reduced in *Cdkl5* +/− and *Cdkl5* −/− females compared to their wild-type counterparts (Figures 7(a)–7(c)). Evaluation of dendritic spine density showed that granule neurons of *Cdkl5* +/− and *Cdkl5* −/− females had reduced spine density [*F*_(2, 8)_ = 9.355, *p* = 0.008; Fisher's LSD: *p* = 0.0445 and *p* = 0.0026, resp.] in comparison with wild-type (+/+) mice (Figures 7(d) and 7(f)). Moreover, separate counts of different classes of dendritic spines revealed that granule neurons of both genotypes had a higher percentage of immature spines (filopodium-like, thin-shaped, and stubby-shaped) and a reduced percentage of mature spines (chi-squared test: *p* = 0.0153 and *p* = 0.0196, resp.) compared to +/+ mice (Figures 7(e) and 7(f)). Since dendritic spine structure is fundamental for synaptic contact maintenance, we further evaluated the number of immunoreactive puncta for PSD-95 (postsynaptic density protein 95) in the molecular layer of the hippocampal dentate gyrus of heterozygous and homozygous *Cdkl5* female mice. *Cdkl5* −/− females displayed a strong reduction in the number of PSD-95-positive puncta [*F*_(2, 8)_ = 30.66, *p* = 0.0002; Fisher's LSD: *p* < 0.0001] compared to *Cdkl5* +/+ female mice (Figures 7(g) and 7(h)), while *Cdkl5* +/− mice showed intermediate levels of PSD-95 puncta (*p* = 0.0007) (Figures 7(g) and 7(h)).

### 3.8. *Cdkl5* +/− and *Cdkl5* −/− Female Mice Exhibit Age-Related Alterations in AKT and ERK Signaling Pathways

**Figure 8:**
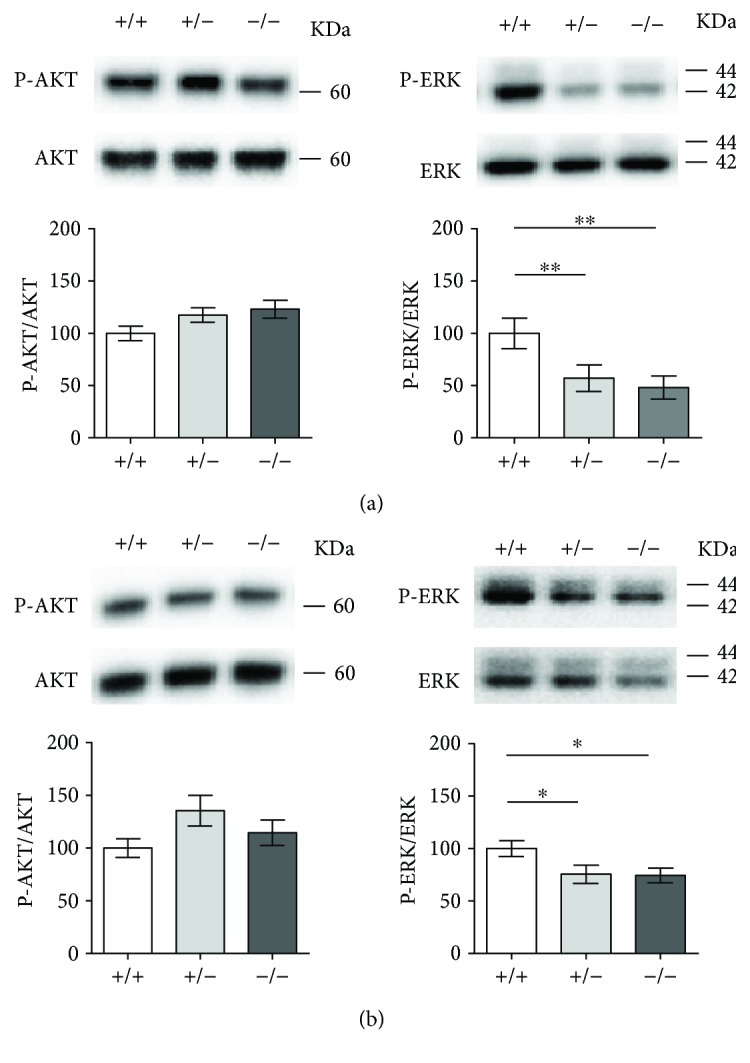
Altered ERK signaling pathway in *Cdkl5* +/− female mice. (a) Western blot analysis of P-AKT-Ser 473 levels normalized to total AKT levels (left histograms) and P-ERK-Ser 42–44 levels normalized to total ERK levels (right histograms) in the hippocampus (a) and cerebellum (b) of *Cdkl5* +/+ (*n* = 6 and *n* = 5, resp.), *Cdkl5* +/− (*n* = 6 and *n* = 8, resp.), and *Cdkl5* −/− (*n* = 6 and *n* = 5, resp.) mice. Immunoblots are examples from one animal of each experimental group. Data are expressed as a percentage of the values of *Cdkl5* +/+ mice. Values are represented as means ± SE. ^∗^ *p* < 0.05 and ^∗∗^ *p* < 0.01 (Fisher's LSD after ANOVA).

We examined whether the AKT signaling pathway, known to be altered in *Cdkl5 KO* male mice [22, 23, 25], is similarly affected in adult *Cdkl5* +/− and *Cdkl5* −/− female mice. Surprisingly, Western blots on hippocampal and cerebellar extracts from adult (3–4 months of age) *Cdkl5* KO females revealed no change in P-AKT-Ser473 levels [*F*_(2, 13)_ = 1.846, *p* = 0.1970 and *F*_(2, 12)_ = 1.460, *p* = 0.2720, resp.] (Figures 8(a) and 8(b)). Likewise, no change in *β*-catenin levels, an AKT downstream target, was observed in hippocampal extracts [*F*_(2, 10)_ = 0.1222, *p* = 0.8863] (Supplementary Figure 1A). Interestingly, decreased levels of phosphorylated ERK1/2 were observed in extracts of the hippocampus [*F*_(2, 13)_ = 7.897, *p* = 0.0057; Fisher's LSD: *p* = 0.0071 and *p* = 0.0027, resp.] and cerebellum [*F*_(2, 17)_ = 4.465, *p* = 0.0276; Fisher's LSD: *p* = 0.0170 and *p* = 0.0191, resp.] from both *Cdkl5* +/− and *Cdkl5* −/− female mice, when compared to their wild-type counterparts (Figures 8(a) and 8(b)). In line with decreased P-ERK1/2 levels, we found decreased levels of phosphorylated MSK1 (P-MSK1-Thr581; mitogen- and stress-activated protein kinase), a ERK1/2 downstream phosphorylation target, in the hippocampus of adult *Cdkl5* +/− and *Cdkl5* −/− mice [*F*_(2, 13)_ = 4.685, *p* = 0.0294; Fisher's LSD: *p* = 0.0385 and *p* = 0.0119, resp.] (Supplementary Figure 1A).

In order to evaluate whether there are age-related changes in AKT and ERK1/2 signal pathways in *Cdkl5* KO mice, we analyzed P-AKT-Ser473, *β*-catenin, P-ERK1/2, and P-MSK1-Thr581 levels in the hippocampus of juvenile (3 week old) and young adult (8 week old) *Cdkl5* KO female mice. In line with previous results [23, 25, 35], we found that juvenile *Cdkl5* +/− and *Cdkl5* −/− mice show reduced P-AKT-Ser473 [*F*_(2, 6)_ = 7.844, *p* = 0.0212; Fisher's LSD: *p* = 0.0253 and *p* = 0.0094, resp.] and *β*-catenin levels [*F*_(2, 10)_ = 6.635, *p* = 0.0147; Fisher's LSD: *p* = 0.0065 and *p* = 0.0169, resp.] (Supplementary Figure 1B) in comparison with their wild-type counterparts. Differently, no changes in P-ERK1/2 [*F*_(2, 10)_ = 1.241, *p* = 0.3299] and P-MSK1-Thr581 [*F*_(2, 12)_ = 0.0720, *p* = 0.9309] levels were observed in juvenile *Cdkl5* KO female mice (Supplementary Figure 1C). Western blots on hippocampal extracts from young adult *Cdkl5* +/− and *Cdkl5* −/− KO females revealed that both genotypes show decreased P-AKT-Ser473 [*F*_(2, 11)_ = 5.076, *p* = 0.0274; Fisher's LSD: *p* = 0.0566 and *p* = 0.0093, resp.] and P-ERK1/2 [*F*_(2, 9)_ = 5.711, *p* = 0.0250; Fisher's LSD: *p* = 0.0174 and *p* = 0.0124, resp.] levels (Supplementary Figure 1E), compared to their wild-type counterparts. In accordance, *β*-catenin [*F*_(2, 13)_ = 6.907, *p* = 0.0090; Fisher's LSD: *p* = 0.0560 and *p* = 0.0026, resp.] (Supplementary Figure 1D) and P-MSK1-Thr581 [*F*_(2, 9)_ = 8.202, *p* = 0.0094; Fisher's LSD: *p* = 0.0110 and *p* = 0.0036, resp.] (Supplementary Figure 1E) levels were reduced in *Cdkl5* +/− and *Cdkl5* −/− female mice.

## 4. Discussion

Animal models of neurological disorders serve as one of the most valuable tools for the development and validation of potential therapies. They enable scientists to test the safety and efficacy of new therapeutic agents *in vivo* and to acquire a detailed understanding of mechanisms of action for new drugs, in order to speed therapies along toward human clinical trials. Since the majority of CDKL5 patients are females with heterozygous CDKL5 mutations, the behavioral and molecular characterization of heterozygous *Cdkl5* KO (*Cdkl5* +/−) female mice is of utmost importance in order to advance preclinical and translational studies. In the present study, we found that heterozygous *Cdkl5* female mice recapitulate several behavioral aspects of CDKL5 disorder including autistic-like behaviors, motor impaired behavior, learning and memory disability, and abnormal breathing pattern. These phenotypes have been described in CDKL5 patients [3, 4, 7] and may mimic the absence of hand skills, the intellectual disability, hyperactivity, poor response to social interactions, and breathing problems that have been described. Heterozygous *Cdkl5* KO female mice, similarly to *Cdkl5* male mice [20, 24, 33–35], show neuroanatomical defects, including dendritic hypotrophy and defects in spine density/maturation. Moreover, we found that loss of Cdkl5 in *Cdkl5* KO female mice impaired, in an age-related manner, phosphorylation levels of the protein kinase B (Akt) and the extracellular signal-regulated kinase (ERK1/2), important signaling mediators in brain function. Therefore, we believe that heterozygous *Cdkl5* KO female mice are a valuable tool for further studies aimed at improving the neurological phenotype due to mutations in CDKL5.

### 4.1. General Health and Sensory Abnormalities

Although no significant difference was observed in body weight among young adult wild-type, heterozygous, and homozygous *Cdkl5* female mice ([23] and present study), we found that adult heterozygous and homozygous *Cdkl5* females showed a lower body weight than did age-matched wild-type females. The decreased weight gain observed in heterozygous and homozygous *Cdkl5* female mice might be secondary to increased energy expenditure, resulting from both an increased basal metabolic rate and locomotor activity. Since olfactory inputs help coordinate food appreciation and selection, but have an important role in energy balance, influencing body weight [43], our finding that heterozygous and homozygous *Cdkl5* female mice showed reduced olfaction could be an explanation for the decreased weight gain. On the other hand, increased locomotor activity and locomotion stereotypes, such as elevated vertical activity (vertical jumps), observed in heterozygous and homozygous *Cdkl5* female mice, could influence body weight by increasing energy expenditure. With regard to reduced olfaction, it remains to be established whether alterations in the neuronal circuitry of the olfactory bulb and/or a reduced neurogenesis underlie this defect in *Cdkl5* KO mice.

### 4.2. Autism-Like Features

Autistic-like (ASD-like) features, including deficits in social interaction, disinterest for the surrounding environment, and stereotypic/repetitive behaviors, have been commonly described in CDKL5 patients [44, 45] and male *Cdkl5* KO mouse models [22, 32]. Our finding that heterozygous and homozygous *Cdkl5* female mice show impaired social behaviors (reduced nest building ability), decreased interest toward the environment (reduced marble burying), and increased stereotypic behaviors (elevated vertical activity in novel environments) suggests that the heterozygous *Cdkl5* female mouse model mirrors the ASD-like features of CDKL5 female patients.

### 4.3. Motor Abnormalities

Motor dysfunction is a prominent feature of CDKL5 deficiency, with the majority of patients having some degree of impairment in gross and fine motor skills, including gait dysfunction and defects in sitting, standing, walking, and grasping [45]. Consistent with this severe motor phenotype in female CDKL5 patients and with previous data in male *Cdkl5* KO mice [22, 23, 31, 32], we found impaired motor coordination, assessed using the accelerating rotarod assay, and novel environment-dependent hyperactivity in heterozygous and homozygous *Cdkl5* KO female mice. Since female *Cdkl5* KO mice had a tendency to ride the rotarod beam around rather than walk forward, in addition to the latency to fall from the rotating rod, we scored the frequency of these passive rotations. While hanging onto the rotating rod is not commonly seen in wild-type mice, we found that both homozygous and heterozygous *Cdkl5* KO female mice showed an increased frequency of uncoordinated rotations, indicating a serious difficulty in motor coordination. *Cdkl5* KO female mice showed higher levels of activity in the open field similar to what has been described in male *Cdkl5* KO mice [22, 32, 35]. This hyperactivity was not related to changes in emotionality/anxiety, as it did not affect the activity in the central part of the field (increased exploration at the edges/walls compared to the center of the arena).

### 4.4. Cognitive Impairments

Severe intellectual disability is a central feature of human CDKL5 deficiency [44]. Impairment in learning and memory is also a consistent feature in male *Cdkl5* KO mice [20, 22, 24, 25, 30, 32, 35]. A marked phenotype of heterozygous and homozygous *Cdkl5* females was attained in the spatial learning and memory paradigm. The acquisition of allocentric learning was markedly impaired in *Cdkl5* KO female mice, thus reinforcing the concept that loss of Cdkl5 is affecting hippocampal learning and memory function. Since learning/memory defects evaluated in the Morris water maze task might be influenced by the differences in swimming speed or in the motivational/motor (floating behavior) aspects observed between wild-type and *Cdkl5* KO female mice, we also tested memory in a passive avoidance test [24]. Again, heterozygous and homozygous *Cdkl5* KO female mice showed a substantial memory impairment (−40%), reinforcing the concept that loss of Cdkl5 leads to alterations in the acquisition process.

### 4.5. Respiratory Problems

To complete our neurobehavioral studies, we monitored breathing pattern, a physiological parameter. Respiratory problems, including the presence and frequency of breathing irregularities (hyperventilation, apnea), are a reported feature in CDKL5 patients [46, 47]. Moreover, disrupted respiratory function and an increased number of apneas during sleep have been recently described also in male *Cdkl5* KO mice [40, 48]. We observed a similar increase in apnea occurrence during NREM sleep and during the whole sleep time in heterozygous and homozygous *Cdkl5* KO female mice, strengthening the statement that the sleep apnea occurrence rate can be a significant biomarker for validation of future therapies in CDKL5 disorder.

### 4.6. Hippocampal Neuroanatomical Defects

Several studies have shown that *Cdkl5* KO male mice, in addition to various behavioral abnormalities, exhibit profound changes in neuronal morphology [20, 22–25, 33–35]. Moreover, these neuroanatomical alterations, including defects in neuronal survival and maturation and defects in the organization of dendritic spines as well as in the density of PSD-95 dendritic clusters, have been shown to be instrumental as preclinical readouts for testing potential therapeutics [20, 24, 33, 35]. Our present findings that heterozygous *Cdkl5* KO females show altered neuronal maturation and synapse development confirm that cellular phenotypes, in addition to behavioral phenotypes, are useful outcome measures in preclinical studies.

### 4.7. Pathway Dysfunctions

In line with previous evidence [23, 25], we found that juvenile *Cdkl5* KO female mice show an alteration in the AKT pathway but no differences in the phosphorylation levels of ERK1/2 in the hippocampal region. Our present findings that young adult (8 week old) *Cdkl5* KO female mice show defects in both AKT and ERK signaling pathways, while adult (3–4 months of age) *Cdkl5* KO females show a strong reduction in ERK1/2 phosphorylation, but no alterations in AKT phosphorylation levels might be explained by age-related changes in the activation and/or modulation of these pathways [49, 50]. Interestingly, AKT is highly expressed in the developing mouse brain but is present at low levels in the adult brain [51]. On the other hand, ERK1/2 is abundant in the adult brain, and its activation can play multiple roles in the activity-dependent regulation of neuronal function [52]. Consistent with its critical role in key cellular activities, dysregulation of ERK signaling has been implicated in the pathogenesis of many neuropsychiatric and neurological disorders including autism [53], mental retardation [54], and several neurodegenerative diseases such as Alzheimer's disease, Parkinson's disease, and amyotrophic lateral sclerosis [55]. Our data, showing age-related changes in AKT and ERK signaling pathways in *Cdkl5* KO mice, indicate that the changes in signaling pathways occurring during the stage of early brain development due to loss of Cdkl5 differ from those that present later on in adulthood. The direct functional effects of these changes on CDKL5 pathogenesis are not yet known. However, the age dependence of these alterations suggests that the efficacy of signaling pathway-targeted therapeutics may be limited to different developmental periods.

### 4.8. Mosaic Cdkl5 Expression and Phenotypic Outcomes

Heterozygous female CDKL5-deficient patients show a vast clinical heterogeneity that spans from milder forms—with less severe epilepsy and less severe comorbidities—to more severe forms featuring intractable seizures and the lack of achievement of developmental milestones [45]. As seen for other dominant X-linked disorders [56], this clinical heterogeneity might be, at least in part, dependent on the phenomenon of variable XCI in heterozygous females. Blood analysis in CDKL5 female patients indicates a random (50%) XCI pattern [44, 57–59], suggesting that different phenotypes are not the result of X-inactivation status. However, XCI skewing is difficult to prove in human brains, since it is frequently measured indirectly from peripheral blood samples and it is difficult to determine whether the same XCI ratio is also present in the brain. Furthermore, the distribution of XCI in the brain may differ from region to region due to its tissue-specific variation [60]. As previously reported [9, 10], we found that Cdkl5 levels vary between the individual brain areas, with the highest Cdkl5 levels in the cortex and hippocampus and lower levels in the cerebellum. In all analyzed brain regions, we found that *Cdkl5* +/− mice show average decreased levels of Cdkl5 to ∼42% of those contained in wild-type counterparts. Our results, which show little variability in Cdkl5 protein expression among the *Cdkl5* +/− brain samples, provide a first indication of balanced XCI patterns in heterozygous *Cdkl5* KO mouse brains. This might explain the relatively small degree of phenotypic variability among heterozygous female mice. Moreover, the putative skewing toward the mutated X chromosome found in the hippocampus of heterozygous females (decreased levels of Cdkl5 ~ 36%) could explain the severe phenotypic outcome in hippocampus-dependent behaviors that is more similar to homozygous *Cdkl5* KO female and hemizygous *Cdkl5* KO male mice (Table 1) [20, 35, 61]. Further studies based on Cdkl5 single-cell based immunodetection in heterozygous brains are required to accurately evaluate whether XCI influences the phenotypic outcome of loss of *Cdkl5* in heterozygous female mice.

By comparing the behavioral results obtained in this study with literature data (Table 1), we found that heterozygous *Cdkl5* KO females develop behavioral abnormalities that are comparable, albeit milder, to defects identified in homozygous *Cdkl5* KO (*Cdkl5* −/−) female and hemizygous *Cdkl5* KO (*Cdkl5* −/Y) male mice. The assessment of specificity and sensitivity of the behavioral tests to discriminate between control and *Cdkl5* KO female mice (ROC analysis; Supplementary Table 1) shows that several behaviors, including autistic-like (i.e., nest building) and cognitive behaviors (i.e., passive avoidance), and brain functions, such as sleep patterns, had AUC values > 0.8, indicating that they might be reliable preclinical readouts for testing the translational potential of therapeutics in females.

Despite the presence of numerous neurobehavioral defects, both *Cdkl5* KO male and female mice do not show spontaneous epilepsy, a hallmark clinical feature of CDKL5 disorder. Even if alterations in the excitatory/inhibitory balance, frequently postulated as a mechanism underlying epileptogenesis and seizure, were observed in *Cdkl5* KO mice [31, 34], it is likely that the differences of neural connectivity between humans and mice might underlie the different seizure susceptibility in the *Cdkl5* KO mouse model.

### 4.9. Conclusions

Recent studies in *Cdkl5* KO mice have revealed different mechanisms that might be therapeutically manipulated and have produced compelling evidence that signaling pathways downstream of CDKL5 can effectively be targeted to ameliorate CDKL5-related symptoms in mice [20, 24, 33]. However, the majority of work involving *Cdkl5* mouse models has focused on the loss of Cdkl5 in male mice, avoiding the potential cofounding effects of variable X-chromosome inactivation (XCI) in female mice. Our study provides a comprehensive overview of neurobehavioral and physiological phenotypes of the heterozygous female *Cdkl5* KO mouse and demonstrates that this mouse model recapitulates multiple features observed in CDKL5 patients; therefore, it might be considered a valuable animal model for future therapeutic studies.

## Figures and Tables

**Table 1:** Comparison of behavioral phenotypes in Cdkl5 KO female and male mice.

		*Cdkl5* +/−	*Cdkl5* −/−	*Cdkl5* −/Y	
Olfactory performance	Buried food test	+++	+++	na	
Autistic-like features	Nest building 22 hours	+++	+++	+++	[22]
	Marble burying	++	+++	na	
Motor function	Rotarod^∗^	+	+	++	[22, 32]
	Open field	+	++	+++	[32]
Learning and memory	Morris water maze learning^∗∗^	++	+++	+++	[20, 24]
	Morris water maze probe	+++	+++	+++	[20, 24]
	Passive avoidance	++	++	+++	[24]
Breathing	Sleep apneas	+++	+++	+++	[40]

Phenotypes are compared to their sex-matched wild-type condition. +, 10–25% of difference; ++, 25–50% of difference; and +++, >50% of difference. na: not assessed. ^∗^Average of all trials; ^∗∗^average of latency day 2–day 5.

## Data Availability

The data used to support the findings of this study are available from the corresponding author upon request.

## Additional Points

*Highlights.* Heterozygous *Cdkl5* KO female mice show autism-like behaviors and defects in motor coordination. Heterozygous *Cdkl5* KO female mice show defects in learning and memory performance. Heterozygous *Cdkl5* KO female mice show breathing abnormalities. Heterozygous *Cdkl5* KO female mice show age-related alterations of the ERK1/2 and AKT pathways. Heterozygous *Cdkl5* KO female mice recapitulate multiple features observed in CDKL5 patients.

## Supplementary Materials

Supplementary 1 Supplementary Figure 1: age-dependent alteration in AKT and ERK signaling pathways in *Cdkl5* +/− and *Cdkl5* −/− female mice. A: Western blot analysis of *β*-catenin levels normalized to GAPDH (glyceraldehyde 3-phosphate dehydrogenase) levels (left histogram) and P-MSK1-Thr 581 levels normalized to GAPDH (right histogram) in the hippocampus of 12–14 week old *Cdkl5* +/+ (*n* = 4 and *n* = 6, resp.), *Cdkl5* +/− (*n* = 5) and *Cdkl5* −/− (*n* = 4 and *n* = 5, resp.) mice. B: Western blot analysis of P-AKT-Ser 473 levels normalized to total AKT levels (left histogram) and *β*-catenin levels normalized to GAPDH levels (right histogram) in the hippocampus of 3 week old *Cdkl5* +/+ (*n* = 3 and *n* = 4, resp.), *Cdkl5* +/− (*n* = 3 and *n* = 4, resp.), and *Cdkl5* −/− (*n* = 3 and *n* = 5, resp.) mice. C: Western blot analysis of P-ERK-Ser 42–44 levels normalized to total ERK levels (left histograms) and P-MSK1-Thr 581 levels normalized to GAPDH (right histogram) in the hippocampus of 3 week old *Cdkl5* +/+ (*n* = 4 and *n* = 5, resp.), *Cdkl5* +/− (*n* = 4 and *n* = 5, resp.), and *Cdkl5* −/− (*n* = 4 and *n* = 5, resp.) mice. D: Western blot analysis of P-AKT-Ser 473 levels normalized to total AKT levels (left histograms) and *β*-catenin levels normalized to GAPDH (right histogram) levels in the hippocampus of 8 week old *Cdkl5* +/+ (*n* = 4 and *n* = 5, resp.), *Cdkl5* +/− (*n* = 4 and *n* = 5, resp.), and *Cdkl5* −/− (*n* = 6) mice. E: Western blot analysis of P-ERK-Ser 42–44 levels normalized to total ERK levels (left histograms) and P-MSK1-Thr 581 levels normalized to GAPDH (right histogram) in the hippocampus of 8 week old *Cdkl5* +/+ (*n* = 3), *Cdkl5* +/− (*n* = 5), and *Cdkl5* −/− (*n* = 4) mice. Immunoblots are examples from one animal of each experimental group. Data are expressed as a percentage of the values of *Cdkl5* +/+ mice. Values are represented as means ± SE. ^∗^ *p* < 0.05 and ^∗∗^ *p* < 0.01 (Fisher's LSD after ANOVA).

Supplementary 2 Supplementary Table 1: ROC analysis on neurobehavioral and functional assays. The relative area under the curve (AUC) and correspondent *p* values are reported. The area under the curve (AUC) reflects the specificity and sensitivity of a test and ranges from totally noninformative (AUC = 0.5) to perfect test (AUC = 1).
